# Role of T Lymphocytes in Type 2 Diabetes and Diabetes-Associated Inflammation

**DOI:** 10.1155/2017/6494795

**Published:** 2017-01-31

**Authors:** Chang Xia, Xiaoquan Rao, Jixin Zhong

**Affiliations:** ^1^College of Health Science & Nursing, Wuhan Polytechnic University, Wuhan, Hubei, China; ^2^Cardiovascular Research Institute, Case Western Reserve University, Cleveland, OH, USA

## Abstract

Although a critical role of adaptive immune system has been confirmed in driving local and systemic inflammation in type 2 diabetes and promoting insulin resistance, the underlying mechanism is not completely understood. Inflammatory regulation has been focused on innate immunity especially macrophage for a long time, while increasing evidence suggests T cells are crucial for the development of metabolic inflammation and insulin resistance since 2009. There was growing evidence supporting the critical implication of T cells in the pathogenesis of type 2 diabetes. We will discuss the available effect of T cells subsets in adaptive immune system associated with the procession of T2DM, which may unveil several potential strategies that could provide successful therapies in the future.

## 1. Introduction

Type 2 diabetes mellitus (T2DM) is characterized by impaired insulin secretion, glucose intolerance, and hyperglycemia. T2DM is widely viewed as a chronic, low-grade inflammatory disease caused by long-term immune system imbalance, metabolic syndrome, or nutrient excess associated with obesity [1, 2]. In addition, T2DM associated complications in the kidneys, arteries, and eyes are also manifested by inflammatory process [3]. Therefore, inflammation is considered as a major driving force in T2DM and associated complications.

Inflammation was first linked to insulin resistance and diabetes in the early 1990s. Hotamisligil et al. reported an increase of TNF-*α* in adipose tissue from different animal models of obesity and diabetes. Neutralization of TNF-*α* in obese rats improved peripheral glucose uptake [4]. Studies have indicated that other inflammatory cytokines such as IL-1*β* and IFN-*γ* which are increased in obesity and diabetes also modulate insulin signaling [5, 6]. On the other hand, a number of anti-inflammatory cytokines, such as IL-4 and IL-10, have been associated with the protection of insulin sensitivity [7]. Macrophages are the major inflammatory cell type in the glucose-utilizing tissues such as adipose tissue and liver. For example, macrophage in the adipose tissue increased from ~5% in lean subjects to a level of up to 50% of all adipose tissue cells in obese individuals [8, 9]. Therefore, early studies on inflammatory regulation of diabetes have been focused on innate immune function. Recent studies, however, suggest adaptive immune system, especially T lymphocyte, also plays a pivotal role in the pathogenesis of T2DM. There has been a rapid growth in our understanding of the role of T cell in the pathogenesis of T2DM in recent years, necessitating a timely review on this topic. In the review, we will discuss the functional involvement of T cells in the pathogenesis of T2DM, especially the regulatory effects of T cells on chronic inflammation.

## 2. Th1 and Th2 Cells

Increasing evidence suggests a pathological role for CD4+ T cells in obesity and insulin resistance. Obesity is a major critical risk factor for T2DM. A recent study by Shirakawa et al. demonstrated that activated CD4+ T cells (CD4^+^CD44^hi^CD62L^lo^) increased in the visceral adipose tissue of obese mice. These cells also expressed PD-1 and CD153 and displayed characteristics of cellular senescence [10]. It has also been shown that obesity induces MHC class II expression on adipocytes and thus activates CD4+ T cells to initiate adipose tissue inflammation [11]. These studies suggest CD4+ T cells may play an important role in obesity and obesity-induced insulin resistance.

CD4+ effector T cells can be further divided into proinflammatory Th1, Th17, and anti-inflammatory Th2 and Foxp3+ regulatory T cell (Treg) subtypes based on their functionality and cytokine production [12]. Once activated, Th1 and Th2 cells show many of the significant signs of inflammation, such as releasing large amount of cytokines. Th1 cells could produce interferon- (IFN-) gamma, interleukin-2 (IL-2), and tumor necrosis factor- (TNF-) beta, triggering cell-mediated immunity and phagocyte-dependent inflammation [12]. Th2 cells, in contrast, produce IL-4, IL-5, IL-6, IL-9, IL-10, and IL-13 to regulate antibody responses [13]. Studies have shown that Th1 and Th2 cells had a key functional role in regulating inflammatory process, although they are activated later than macrophages in inflammation [14–16].

It is widely accepted that macrophage activation is affected by T cells. T cells play a dominant role in promoting or sustaining inflammatory processes and insulin resistance through inducing proinflammatory cytokines in metabolic organs, such as the adipose tissue, liver, muscle, and pancreas. Macrophages are the major inflammatory cells in the adipose tissue in obesity, increasing from 5–10% in lean subjects to up to 50% in obese individuals [8, 9]. Based on their cytokine production and activation conditions, adipose tissue macrophages are categorized into two populations, proinflammatory M1 (classically activated macrophage) and anti-inflammatory M2 (alternatively activated macrophage). M1 macrophages release proinflammatory cytokines including TNF-*α*, IL-6, and IL-1*β*, which contribute to the local and systemic inflammation [16]. In contrast, M2 macrophages secrete IL-10, which could inhibit the activity of most proinflammatory cell types including M1 macrophages [16, 17]. IL-10 has been shown to suppress TNF-*α* by interacting with the p38/MAPK pathway [17, 18]. Th1 cells could secret IFN-*γ* to promote M1 polarization and enhance its proinflammatory functions by inducing the release of IL-1, IL-6, and TNF-*α*. In contrast, Th2 cells could produce anti-inflammatory IL-4 and IL-13 to skew the differentiation of macrophage towards M2 [19, 20]. In diet-induced obesity, CD4+T cells were increased in the adipose tissue and induced the recruitment and differentiation of TNF-*α*-secreting M1, while the number of IL-10-secreting M2 reduced in the adipose tissue [21]. These findings suggested the macrophage polarization of proinflammatory M1 versus anti-inflammatory M2 was viewed as a key factor in the development of obesity and T2DM [17]. Therefore, Th1 and Th2 responses, which are closely related to M1/M2 polarization, may also have a critical role in obesity and T2DM.

Several clinical studies have confirmed that Th1 was upregulated in the adipose tissue and peripheral blood from the individuals with prediabetes or T2DM [22]. Zeng and coworkers reported an imbalance of CD4+ T helper cell subsets including Treg, Th1, and Th17 in the patients with T2DM [23]. In contrast, naive CD4+ T cells were reported to decline in the patients with T2DM, which may be associated with adaptive immune activation and chronic inflammation during the pathogenesis of T2DM [24]. Similar findings were observed in lymphocyte-deficient mice, which displayed a less severe phenotype of insulin resistance on short-term high-fat diet [25]. Th1 and CD8+ lymphocytes in the adipose tissue were significantly increased in response to a high-fat diet, while anti-inflammatory Th2 and Treg cells were decreased [26].

CD4+ helper T cells also play a critical role in a series of complications associated with T2DM. Activated T lymphocytes and the inflammatory cytokines increased in the kidneys in patients with T2DM [27, 28]. Experimental evidence indicates that the activation of Th2 cell-mediated immunity is delayed and impaired in diabetes [29]. It has been shown that Th1-associated cytokines induce hyperinflammatory response and subsequently lead to progressive innate immune response [30]. It is reported that the circulating levels of Th1-associated cytokines increased in diabetic patients [30]. The levels of T cell-related cytokines such as IL-10 and IL-17 were significantly higher in patients with T2DM, suggesting an involvement of T cells in diabetes [31]. In consistency, oral anti-CD3 plus glucosylceramide (an NKT cell target antigen) treatment was shown to induce the production of IL-10 and TGF-*β*, which was associated with improved fasting glucose, visceral adipose tissue inflammation, liver enzymes, and hepatic steatosis in ob/ob mice [32]. These findings suggest both Th1 and Th2 are closely associated with insulin resistance and chronic inflammation in T2DM.

## 3. Th17 Cells

Th17, an important proinflammatory CD4+ T cell subtype secreting IL-17, has also been associated with T2DM [33, 34]. A recent study examined the differentiation of different CD4+ T cell subsets in T2DM by analyzing the cytokine production by PBMCs [35]. The results demonstrated that Th17 cells increased in T2DM patients and might be associated with dysregulated lipid metabolism [35]. Studies have shown that IL-17 could stimulate the production of TNF-*α*, the first cytokine being associated with obesity and insulin resistance. It is reported that intestinal Th17 cells may induce AMPs, specifically Reg3b and Reg3g, contributing to increased intestinal permeability in high-fat diet-fed mice [35, 36]. Th17 has also been shown to increase in diabetic complications such as diabetic nephropathy [28]. The function of Th17 cells could not be ignored in the progress of complications associated with T2DM. The result showed that Th17 counts and Th17/Treg ratio increased in diabetic nephropathy patients compared with healthy individuals and diabetic patients without nephropathy [28]. The adipose tissue (AT) DCs isolated from obese animals and humans were associated with the differentiation of Th17 cells in vitro [37]. However, the interaction between DCs and Th17 cells have not yet been identified in AT from lean or obese animals [38]. Whether CD103 DCs can regulate obesity-induced inflammation in AT by influencing the balance of Treg and Th17 cell differentiation in vivo remains unknown [38].

IL-22 is produced by Th17, the second product downstream of IL-6 and IL-23. It was shown that the microphages from AT could express the IL-22 receptor (IL-22R) and respond to IL-22 to secret more IL-1b, promoting AT inflammation [39, 40]. It is interesting whether IL-22 associated metabolic effects in mice could apply to humans. Both IL-17 and IL- 22 indicate that Th17 may play an alternative role of “protective” and “destructive” in T2DM development [41].

## 4. Regulatory T Cells

Treg cells, a small subset of T lymphocytes constituting only 5–20% of the CD4+ compartment, are thought to be important to prevent excessive inflammatory responses and limit tissue impairment [42, 43]. Typically, they regulate the response of other T cell subtypes but can also influence the activities of innate immune cells [44, 45]. Treg cells are characterized by high-level expression of a fork head/winged-helix transcription factor, Foxp3. Some studies showed that the relative proportion of Th1 to Foxp3+ cells was positively correlated with body mass index (BMI) [46]. A higher proportion of Treg was observed in lean mice [19, 46]. Fat-resident regulatory T cells were commonly observed in close proximity to monocytes or macrophages, which imply potential links with fat associated monocytes or macrophage [19]. The CD62L+ CD44− naive CD4+ T cells were increased in aged fat-specific Treg knockout mice, but there were no significant differences in the levels of inflammatory cytokines in serum, such as TNF-*α*, IL1*β*, IL6, IFN-*γ*, and IL17, compared with wild type mice [19]. Treg cells were higher in the fat versus spleen and lung detected by intracellular staining for IL-10 protein [19]. Because the expression of IL-10 receptor was upregulated in fat Treg cells, it seems the Treg cells not only produced IL-10 but also responded to it [19].

In T2DM, Treg cells could suppress Th1, Th2, and Th17 response to improve insulin resistance. Treg could inhibit the inflammatory response by various pathways, such as surpassing cytokine secretion, modulating the microenvironment, and changing the expression of surface receptors [47]. The appropriate balance between proinflammatory (Th17 or Th1) and anti-inflammatory (Treg) subsets of T cells is vital to maintain host immunity and control inflammatory damage [48]. It was found that the amount of Treg cells decreased in patients with T2DM [46]. Zeng et al. also reported that Treg/Th17 ratio and Treg/Th1 ratio decreased in patients with T2DM [49]. Further analysis suggests peripheral induced Treg but not natural Treg produced in the thymus reduced in T2DM, possibly due to decreased bcl2/bax and low HDL level [49].

Previous study has demonstrated that the expression of CD39, an ectoenzyme highly expressed in Treg cells, increased in CD4+ lymphocytes from T2DM and obese patients [50]. Adenosine triphosphate could be hydrolyzed by CD39 and adenosine diphosphate into adenosine monophosphate (AMP), which subsequently converted into a T cell suppressive metabolite adenosine by CD73 [50]. CD39 was confirmed to be associated with the stability and the suppressive function of Foxp3+Treg cells. T2DM patients with obesity showed a significantly lower level of CD39^hi^ Treg compared with overweight controls [51]. It was reported that PD-L1, CD25, and cytotoxic T lymphocyte-associated protein-4 (CTLA-4) levels were higher on the surface of CD39^hi^ Tregs, compared with CD39^low^ Tregs [51]. In addition, CD39^hi^ Tregs could produce more IL-10 than CD39^low^ Tregs [51]. Adenosine and its analogues have also been demonstrated to promote lymphocyte apoptosis through the A2A receptor, indicating Tregs in diabetic patients may be more susceptible to apoptosis [52].

## 5. CD8+ Cytotoxic T Cells

The inflammation of adipose tissue is considered as a key event leading to the metabolic syndrome, diabetes, and atherosclerotic cardiovascular disease. It was reported that, within two weeks of high-fat diet feeding, the CD8+CD4− T cells were significantly increased in C57BL/6 mice [21]. The amount of CD8+CD4− T cells kept increasing until 15 weeks. In contrast, Treg cells and CD4+ T cells were reduced upon high-fat diet [21]. Unlike in wild type mice, there were no significant increases in the M1 or M2 macrophage fraction in CD8-deficient mice under high-fat diet although both body weight and epididymal fat mass significantly increased on high-fat diet [21]. More importantly, it was shown that CD8+ T cells were essential for induction of macrophage activation and migration to adipose tissue by secreting MCP-1, MCP-3, and RANTES (regulated on activation, normal T cell expressed and secreted) [21, 53]. These studies indicate that CD8+ T cells were crucially involved in evoking inflammatory cascades in obese adipose tissue.

Recent clinical studies also reported that the level of IFN-*γ* produced by CD3+ T cells was positively correlated with BMI in patients with T2DM. Another research indicates that CD8+ T cells increased in both the small bowel and colon in some obese individuals, compared with lean humans [54]. This study also showed the level of IFN-*γ* produced by CD8+ T cells increased in obese individuals [54]. The increased intraepithelial CD8+ T cells may have the potential to modulate the insulin sensitivity of enterocyte [54].

CD137-CD137L interaction contributes to CD8+ cytotoxic T cell proliferation and secretion of IFN-*γ*, TNF-*α*, IL-2, and IL-4 [55, 56]. The expression of CD137 was upregulated in obese humans and mice [56]. CD137-CD137L could promote monocyte and T cell recruitment to the adipose tissue [57]. The inflammation in adipose tissue decreased in CD137^−/−^ mice, but glucose tolerance was strengthened [58]. Therefore, CD137 contributes to abnormal glucose and lipid metabolism, which may involve the expansion and activation of CD8+ T cells.

## 6. **γδ** T Cells

T cells are subdivided into two major populations according to their surface expression of *αβ* and *γδ* T cell receptors (TCR). Activated *γδ* T cells could produce a vast variety of cytokines such as IFN-*γ* and TNF-*α* to regulate the function of other immune cells. In recent years, there is increasing evidence indicating that *γδ* T cells may interact with macrophages, CTLs, Th1/Th2 cells, Treg, and Th17 cells depending on specific microenvironment, bridging innate and adaptive immunity. *γδ* T cells could produce IL-10 and IL-17 [59]. In addition, they also secret TNF-*α* to regulate the activation of CD8+ T cells [60].

Obesity is an important risk factor for chronic inflammatory diseases, such as T2DM and cardiovascular disease [61]. *γδ* T cells have been suggested to play a critical role in chronic inflammatory disease. It was found that obese patients exhibited a decreased level of V*γ*9V*δ*2 T cells and the level of secreting IFN-*γ* was also declined. These V*γ*9V*δ*2 T cells could prefer to differentiate mature T effecter memory cluster of differentiation 45RA (CD45RA+) cells, indicating *γδ* T cells are also involved in inflammation in obesity and diabetes [62]. A recent study reported that IL-2 treatment rescued V*γ*9V*δ*2 T cell cytokine production [63], which provides a novel insight into the pathogenic role *γδ* T cells in the development of T2DM.

## 7. iNKT Cells

Previous studies have demonstrated that circulating natural killer cells (iNKT cells) were decreased and their function was suppressed in obese individuals, compared with lean individuals [64]. It has been identified that iNKT cells are enriched in human and murine adipose tissue [65, 66]. iNKT cells could recognize lipid antigens presented by CD1d but not peptide through MHC molecules in the innate immune system [67, 68]. Further investigation has been shown that adipose iNKT cells are a tissue resident with little influx from the circulation and play an important anti-inflammatory regulatory role in congenic parabiotic mice [69]. However, adipose iNKT cells are obviously decreased in obesity [65, 66, 70]. iNKT cells can regulate the cross talk between innate and adaptive immunity as potent transactivators. Most studies reported that iNKT cell-deficient mice have worse metabolic disorder and weight gain [65, 66, 70, 71]. *α*-Galactosylceramide (*α*GalCer), a potent lipid ligand, could activate iNKT cell. It is shown that injection of *α*GalCer in obesity increases the iNKT cell amount and induces weight loss rapidly, anti-inflammatory macrophage differentiation, and reversal of glucose and insulin sensitivity without hypoglycemia [72, 73]. The majority of findings suggest that iNKT cells in AT play a critical role in regulating local inflammation and protecting against metabolic disorder in obesity [74].

## 8. Conclusion

In conclusion, there is increasing evidence linking the activation of T lymphocytes to T2DM. Due to the importance of inflammation in the development of insulin resistance, T2DM is now considered as an autoimmune disease [75]. Understanding the role of specific immune cells and proinflammatory molecules in obesity and diabetes is required for developing novel therapeutic approaches to modulate metabolic inflammation and insulin resistance.

## References

[1] Guzmán-Flores J. M., López-Briones S. (2012). Cells of the innate and adaptive immunity in type 2 diabetes mellitus and obesity. *Gaceta Medica de Mexico*.

[2] Shu C. J., Benoist C., Mathis D. (2012). The immune system's involvement in obesity-driven type 2 diabetes. *Seminars in Immunology*.

[3] Guarner V., Rubio-Ruiz M. E. (2014). Low-grade systemic inflammation connects aging, metabolic syndrome and cardiovascular disease. *Aging and Health—A Systems Biology Perspective*.

[4] Hotamisligil G. S., Shargill N. S., Spiegelman B. M. (1993). Adipose expression of tumor necrosis factor-*α*: direct role in obesity-linked insulin resistance. *Science*.

[5] Mathis D. (2013). Immunological goings-on in visceral adipose tissue. *Cell Metabolism*.

[6] Ouchi N., Parker J. L., Lugus J. J., Walsh K. (2011). Adipokines in inflammation and metabolic disease. *Nature Reviews Immunology*.

[7] Odegaard J. I., Ricardo-Gonzalez R. R., Goforth M. H. (2007). Macrophage-specific PPAR*γ* controls alternative activation and improves insulin resistance. *Nature*.

[8] Weisberg S. P., McCann D., Desai M., Rosenbaum M., Leibel R. L., Ferrante A. W. (2003). Obesity is associated with macrophage accumulation in adipose tissue. *Journal of Clinical Investigation*.

[9] Boutens L., Stienstra R. (2016). Adipose tissue macrophages: going off track during obesity. *Diabetologia*.

[10] Shirakawa K., Yan X., Shinmura K. (2016). Obesity accelerates T cell senescence in murine visceral adipose tissue. *Journal of Clinical Investigation*.

[11] Deng T., Lyon C. J., Minze L. J. (2013). Class II major histocompatibility complex plays an essential role in obesity-induced adipose inflammation. *Cell Metabolism*.

[12] Raphael I., Nalawade S., Eagar T. N., Forsthuber T. G. (2015). T cell subsets and their signature cytokines in autoimmune and inflammatory diseases. *Cytokine*.

[13] Kahn S. E., Hull R. L., Utzschneider K. M. (2006). Mechanisms linking obesity to insulin resistance and type 2 diabetes. *Nature*.

[14] Martinez F. O. (2008). Macrophage activation and polarization. *Frontiers in Bioscience*.

[15] Cintra D. E., Pauli J. R., Araújo E. P. (2008). Interleukin-10 is a protective factor against diet-induced insulin resistance in liver. *Journal of Hepatology*.

[16] Lumeng C. N., Bodzin J. L., Saltiel A. R. (2007). Obesity induces a phenotypic switch in adipose tissue macrophage polarization. *Journal of Clinical Investigation*.

[17] Winer S., Chan Y., Paltser G. (2009). Normalization of obesity-associated insulin resistance through immunotherapy. *Nature Medicine*.

[18] McGillicuddy F. C., Chiquoine E. H., Hinkle C. C. (2009). Interferon *γ* attenuates insulin signaling, lipid storage, and differentiation in human adipocytes via activation of the JAK/STAT pathway. *The Journal of Biological Chemistry*.

[19] Feuerer M., Herrero L., Cipolletta D. (2009). Lean, but not obese, fat is enriched for a unique population of regulatory T cells that affect metabolic parameters. *Nature Medicine*.

[20] Chatzigeorgiou A., Karalis K. P., Bornstein S. R., Chavakis T. (2012). Lymphocytes in obesity-related adipose tissue inflammation. *Diabetologia*.

[21] Nishimura S., Manabe I., Nagasaki M. (2009). CD8+ effector T cells contribute to macrophage recruitment and adipose tissue inflammation in obesity. *Nature Medicine*.

[22] McLaughlin T., Liu L., Lamendola C. (2014). T-cell profile in adipose tissue is associated with insulin resistance and systemic inflammation in humans. *Arteriosclerosis, Thrombosis, and Vascular Biology*.

[23] Zeng C., Shi X., Zhang B. (2012). The imbalance of Th17/Th1/Tregs in patients with type 2 diabetes: relationship with metabolic factors and complications. *Journal of Molecular Medicine*.

[24] Nekoua M. P., Fachinan R., Atchamou A. K. (2016). Modulation of immune cells and Th1/Th2 cytokines in insulin-treated type 2 diabetes mellitus. *African Health Sciences*.

[25] Lee Y. S., Li P., Huh J. Y. (2011). Inflammation is necessary for long-term but not short-term high-fat diet-induced insulin resistance. *Diabetes*.

[26] Harford K. A., Reynolds C. M., McGillicuddy F. C., Roche H. M. (2011). Fats, inflammation and insulin resistance: insights to the role of macrophage and T-cell accumulation in adipose tissue. *Proceedings of the Nutrition Society*.

[27] Navarro J. F., Mora C. (2006). Diabetes, inflammation, proinflammatory cytokines, and diabetic nephropathy. *TheScientificWorldJournal*.

[28] Abouzeid S., Sherif N. (2015). Role of alteration in Treg/Th17 cells’ balance in nephropathic patients with Type 2 diabetes mellitus. *Electronic physician*.

[29] Wu D., Molofsky A. B., Liang H.-E. (2011). Eosinophils sustain adipose alternatively activated macrophages associated with glucose homeostasis. *Science*.

[30] Francisco C., Catai A., Moura-Tonello S. (2016). Cytokine profile and lymphocyte subsets in type 2 diabetes. *Brazilian Journal of Medical and Biological Research*.

[31] Mocellin S., Marincola F., Rossi C. R., Nitti D., Lise M. (2004). The multifaceted relationship between IL-10 and adaptive immunity: putting together the pieces of a puzzle. *Cytokine and Growth Factor Reviews*.

[32] Ilan Y., Maron R., Tukpah A.-M. (2010). Induction of regulatory T cells decreases adipose inflammation and alleviates insulin resistance in ob/ob mice. *Proceedings of the National Academy of Sciences of the United States of America*.

[33] Zhang C., Xiao C., Wang P. (2014). The alteration of Th1/Th2/Th17/Treg paradigm in patients with type 2 diabetes mellitus: relationship with diabetic nephropathy. *Human Immunology*.

[34] Zuniga L. A., Shen W., Joyce-Shaikh B. (2010). IL-17 regulates adipogenesis, glucose homeostasis, and obesity. *The Journal of Immunology*.

[35] Garidou L., Pomié C., Klopp P. (2015). The gut microbiota regulates intestinal CD4 T cells expressing ROR*γ*t and controls metabolic disease. *Cell Metabolism*.

[36] Hamilton M. K., Boudry G., Lemay D. G., Raybould H. E. (2015). Changes in intestinal barrier function and gut microbiota in high-fat diet-fed rats are dynamic and region dependent. *American Journal of Physiology—Gastrointestinal and Liver Physiology*.

[37] Bertola A., Ciucci T., Rousseau D. (2012). Identification of adipose tissue dendritic cells correlated with obesity-associated insulin-resistance and inducing Th17 responses in mice and patients. *Diabetes*.

[38] Lee B.-C., Lee J. (2014). Cellular and molecular players in adipose tissue inflammation in the development of obesity-induced insulin resistance. *Biochimica et Biophysica Acta—Molecular Basis of Disease*.

[39] Dalmas E., Venteclef N., Caer C. (2014). T cell-derived IL-22 amplifies IL-1*β*-driven inflammation in human adipose tissue: relevance to obesity and type 2 diabetes. *Diabetes*.

[40] Zhao R., Tang D., Yi S. (2014). Elevated peripheral frequencies of Th22 cells: a novel potent participant in obesity and type 2 diabetes. *PLoS ONE*.

[41] Ip B. C., Hogan A. E., Nikolajczyk B. S. (2015). Lymphocyte roles in metabolic dysfunction: of men and mice. *Trends in Endocrinology and Metabolism*.

[42] Zheng Y., Rudensky A. Y. (2007). Foxp3 in control of the regulatory T cell lineage. *Nature Immunology*.

[43] Sakaguchi S., Yamaguchi T., Nomura T., Ono M. (2008). Regulatory T cells and immune tolerance. *Cell*.

[44] Murphy T. J., Choileain N. N., Zang Y., Mannick J. A., Lederer J. A. (2005). CD4^+^CD25^+^ regulatory T cells control innate immune reactivity after injury. *Journal of Immunology*.

[45] Nguyen L. T., Jacobs J., Mathis D., Benoist C. (2007). Where FoxP3-dependent regulatory T cells impinge on the development of inflammatory arthritis. *Arthritis & Rheumatism*.

[46] Jagannathan-Bogdan M., McDonnell M. E., Shin H. (2011). Elevated proinflammatory cytokine production by a skewed T cell compartment requires monocytes and promotes inflammation in type 2 diabetes. *Journal of Immunology*.

[47] Guzmán-Flores J. M., Portales-Pérez D. P. (2013). Mechanisms of suppression by regulatory T cells (Treg). *Gaceta Medica de Mexico*.

[48] Gregor M. F., Hotamisligil G. S. (2011). Inflammatory mechanisms in obesity. *Annual Review of Immunology*.

[49] Zeng L., Lu H., Deng H., Mu P., Li X., Wang M. (2012). Noninferiority effects on glycemic control and *β*-cell function improvement in newly diagnosed type 2 diabetes patients: basal insulin monotherapy versus continuous subcutaneous insulin infusion treatment. *Diabetes Technology and Therapeutics*.

[50] Della Latta V., Cabiati M., Rocchiccioli S., Del Ry S., Morales M.-A. (2013). The role of the adenosinergic system in lung fibrosis. *Pharmacological Research*.

[51] Gu J., Ni X., Pan X. (2016). Human CD39hi regulatory T cells present stronger stability and function under inflammatory conditions. *Cellular & Molecular Immunology*.

[52] Sun Y., Liu W.-Z., Liu T., Feng X., Yang N., Zhou H.-F. (2015). Signaling pathway of MAPK/ERK in cell proliferation, differentiation, migration, senescence and apoptosis. *Journal of Receptors and Signal Transduction*.

[53] Kintscher U., Hartge M., Hess K. (2008). T-lymphocyte infiltration in visceral adipose tissue: a primary event in adipose tissue inflammation and the development of obesity-mediated insulin resistance. *Arteriosclerosis, Thrombosis, and Vascular Biology*.

[54] Luck H., Tsai S., Chung J. (2015). Regulation of obesity-related insulin resistance with gut anti-inflammatory agents. *Cell Metabolism*.

[55] Vinay D. S., Kwon B. S. (1998). Role of 4-1BB in immune responses. *Seminars in Immunology*.

[56] Tu T. H., Kim C.-S., Kang J.-H. (2014). Levels of 4-1BB transcripts and soluble 4-1BB protein are elevated in the adipose tissue of human obese subjects and are associated with inflammatory and metabolic parameters. *International Journal of Obesity*.

[57] Tu T. H., Kim C.-S., Goto T., Kawada T., Kim B.-S., Yu R. (2012). 4-1BB/4-1BBL interaction promotes obesity-induced adipose inflammation by triggering bidirectional inflammatory signaling in adipocytes/macrophages. *Mediators of Inflammation*.

[58] Kim C. S., Kim J. G., Lee B. J. (2011). Deficiency for costimulatory receptor 4-1BB protects against obesity-induced inflammation and metabolic disorders. *Diabetes*.

[59] Rhodes K. A., Andrew E. M., Newton D. J., Tramonti D., Carding S. R. (2008). A subset of IL-10-producing *γδ* T cells protect the liver from *Listeria*-elicited, CD8^+^ T cell-mediated injury. *European Journal of Immunology*.

[60] Jensen K. D. C., Su X., Shin S. (2008). Thymic selection determines *γδ* T cell effector fate: antigen-naive cells make interleukin-17 and antigen-experienced cells make interferon *γ*. *Immunity*.

[61] Lackey D. E., Olefsky J. M. (2016). Regulation of metabolism by the innate immune system. *Nature Reviews Endocrinology*.

[62] Dimova T., Brouwer M., Gosselin F. (2015). Effector V*γ*9V*δ*2 T cells dominate the human fetal *γδ* T-cell repertoire. *Proceedings of the National Academy of Sciences*.

[63] Costanzo A. E., Taylor K. R., Dutt S., Han P. P., Fujioka K., Jameson J. M. (2015). Obesity impairs *γδ* T cell homeostasis and antiviral function in humans. *PLoS ONE*.

[64] Lynch L. A., O'Connell J. M., Kwasnik A. K., Cawood T. J., O'Farrelly C., O'Shea D. B. (2009). Are natural killer cells protecting the metabolically healthy obese patient?. *Obesity*.

[65] Lynch L., O'Shea D., Winter D. C., Geoghegan J., Doherty D. G., O'Farrelly C. (2009). Invariant NKT cells and CD1d^+^ cells amass in human omentum and are depleted in patients with cancer and obesity. *European Journal of Immunology*.

[66] Lynch L., Nowak M., Varghese B. (2012). Adipose tissue invariant NKT cells protect against diet-induced obesity and metabolic disorder through regulatory cytokine production. *Immunity*.

[67] Brigl M., Brenner M. B. (2004). CD1: antigen presentation and T cell function. *Annual Review of Immunology*.

[68] Beckman E. M., Porcelli S. A., Morita C. T., Behar S. M., Furlong S. T., Brenner M. B. (1994). Recognition of a lipid antigen by GDI-restricted *αβ*+ T cells. *Nature*.

[69] Lynch L., Michelet X., Zhang S. (2015). Regulatory *i*NKT cells lack expression of the transcription factor PLZF and control the homeostasis of T_reg_ cells and macrophages in adipose tissue. *Nature Immunology*.

[70] Schipper H. S., Rakhshandehroo M., Van De Graaf S. F. J. (2012). Natural killer T cells in adipose tissue prevent insulin resistance. *Journal of Clinical Investigation*.

[71] Huh J. Y., Kim J. I., Park Y. J. (2013). A novel function of adipocytes in lipid antigen presentation to iNKT cells. *Molecular and Cellular Biology*.

[72] Hams E., Locksley R. M., McKenzie A. N. J., Fallon P. G. (2013). Cutting edge: IL-25 elicits innate lymphoid type 2 and type II NKT cells that regulate obesity in mice. *The Journal of Immunology*.

[73] Ji Y., Sun S., Xu A. (2012). Activation of natural killer T cells promotes M2 macrophage polarization in adipose tissue and improves systemic glucose tolerance via interleukin-4 (IL-4)/STAT6 protein signaling axis in obesity. *Journal of Biological Chemistry*.

[74] Kohlgruber A., Lynch L. (2015). Adipose tissue inflammation in the pathogenesis of type 2 diabetes. *Current Diabetes Reports*.

[75] Tsai S., Clemente-Casares X., Revelo X. S., Winer S., Winer D. A. (2015). Are obesity-related insulin resistance and type 2 diabetes autoimmune diseases?. *Diabetes*.

